# Rapid deterioration of steatotic liver disease due to portal vein stenosis after pancreaticoduodenectomy

**DOI:** 10.1007/s12328-024-02027-5

**Published:** 2024-08-15

**Authors:** Mineto Ohta, Rikiya Kanba, Keisuke Fukushima, Kazutomi Takahashi, Hiroyasu Nishimaki, Tatsuya Sasaki, Ai Fujita, Maika Kanno, Yuki Ogasawara, Kenji Namiki

**Affiliations:** 1 Department of Surgery, Osaki Citizen Hospital, 3-8-1 Honami, Osaki, Miyagi 989-6183 Japan; 2 Department of General Medicine, Osaki Citizen Hospital, 3-8-1 Honami, Osaki, Miyagi 989-6183 Japan; 3 Department of Nutrition, Osaki Citizen Hospital, 3-8-1 Honami, Osaki, Miyagi 989-6183 Japan

**Keywords:** Steatotic liver disease, Pancreatoduodenectomy, Portal vein stenosis

## Abstract

Steatotic liver disease after pancreatoduodenectomy occurs due to various factors, such as exocrine pancreatic insufficiency, impaired intestinal absorption, and malnutrition. The mechanism of steatogenesis differs to that of conventional steatotic liver disease associated with obesity and insulin resistance. We experienced a rare case of rapidly progressive steatotic liver disease accompanied by portal vein stenosis in the early postoperative period after subtotal stomach-preserving pancreaticoduodenectomy for distal cholangiocarcinoma. Although there was a complication due to postoperative drain infection, the patient was discharged from hospital with no nutritional problems. Two months postoperatively, the patient presented to the emergency room with dyspnea. CT showed a markedly steatotic liver, ascites, and portal vein stenosis. A portal vein stent was inserted transhepatically and the steatotic liver disease gradually improved. During the postoperative course, there were no problems indicated by nutritional markers; although the patient had diarrhea associated with postoperative pancreatic exocrine insufficiency, the symptoms were mild and improved after administration of oral pancrelipase. Before the intervention, the patient had intestinal edema, exacerbation of diarrhea, and a low serum zinc concentration, suggesting that impaired absorption caused by intestinal blood stasis and gut barrier dysfunction contributed to the development of steatotic liver disease.

## Introduction

Metabolic dysfunction-associated steatotic liver disease (MASLD) is the most common liver disease worldwide [1]. MASLD is closely associated with obesity, lipodystrophy, insulin resistance syndrome, and metabolic syndrome [2]. The global estimated prevalence of MASLD is 32% and the condition is a leading cause of liver-related morbidity and mortality [3]. It is well-known that steatotic liver disease (SLD) develops after pancreaticoduodenectomy (PD), with a reported incidence of 15–40% [4–6]. However, the steatogenesis that occurs after PD is thought to differ from conventional MASLD. The reported risk factors for post-PD steatogenesis are pancreatic cancer, high preoperative hemoglobin A1c and carbohydrate antigen 19–9 concentrations, large pancreatic resection volume, pancreatic stiffness, female sex, high body mass index, pancreatic leakage, postoperative pancreatic exocrine insufficiency, postoperative impaired intake, and diarrhea [7]. However, the mechanism of MASLD after PD has not yet been elucidated.

We herein present a case of a patient who had undergone subtotal stomach-preserving pancreaticoduodenectomy and at 2 months after surgery developed MASLD accompanied by portal vein (PV) stenosis that rapidly worsened. The patient had several risk factors for MASLD, such as female sex, a hard pancreas, and postoperative diarrhea; however, the time of onset, symptoms, and course of the disease were unusual. The SLD soon improved after PV stenting.

## Case report

**Fig. 1:**
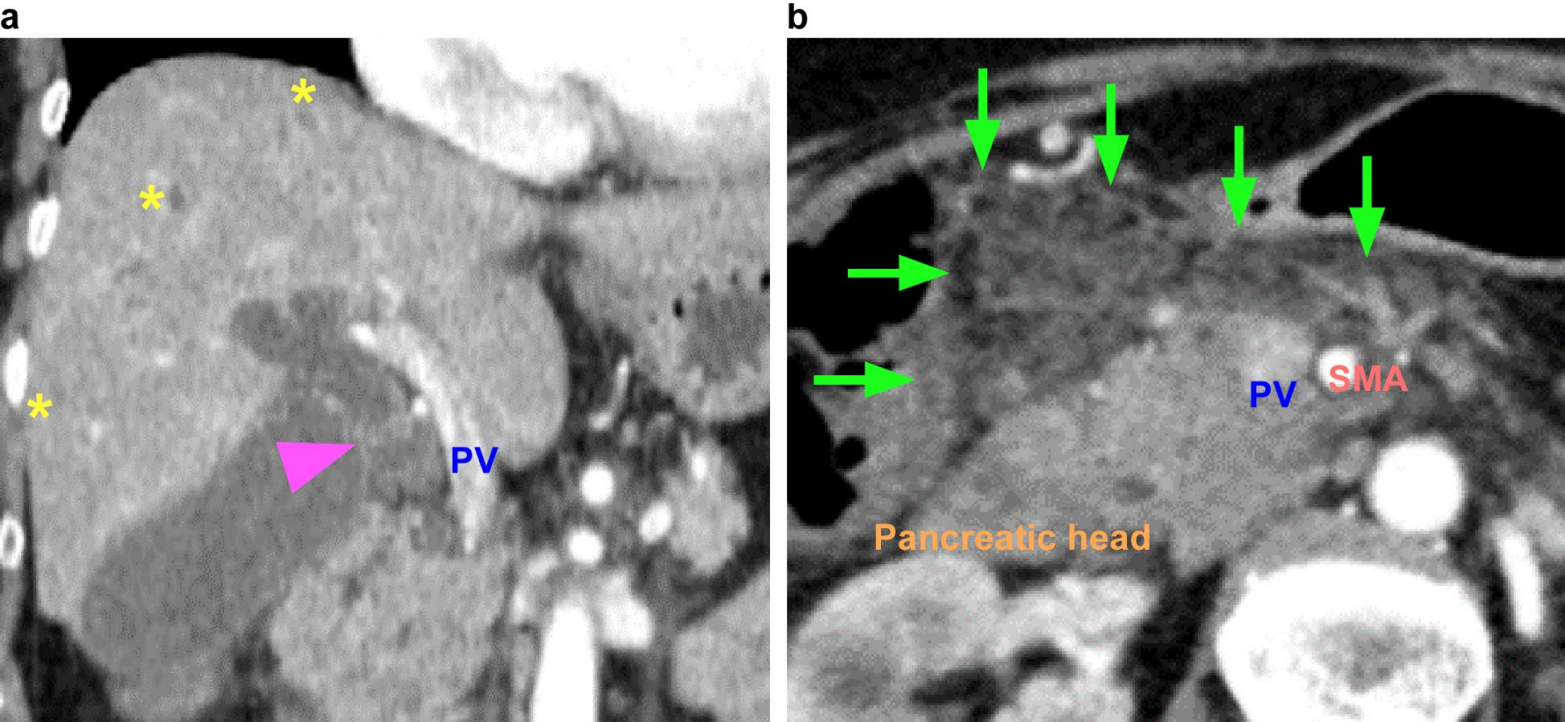
CT images of the tumor and pancreatitis after endoscopic retrograde cholangiopancreatography. **a** Enhanced tumor (arrowhead) is seen in a distal bile duct. The intrahepatic bile duct is mildly dilated (+). **b** Extensive inflammation (arrows) is present from the pancreatic head to the mesentery of the transverse colon

**Fig. 2:**
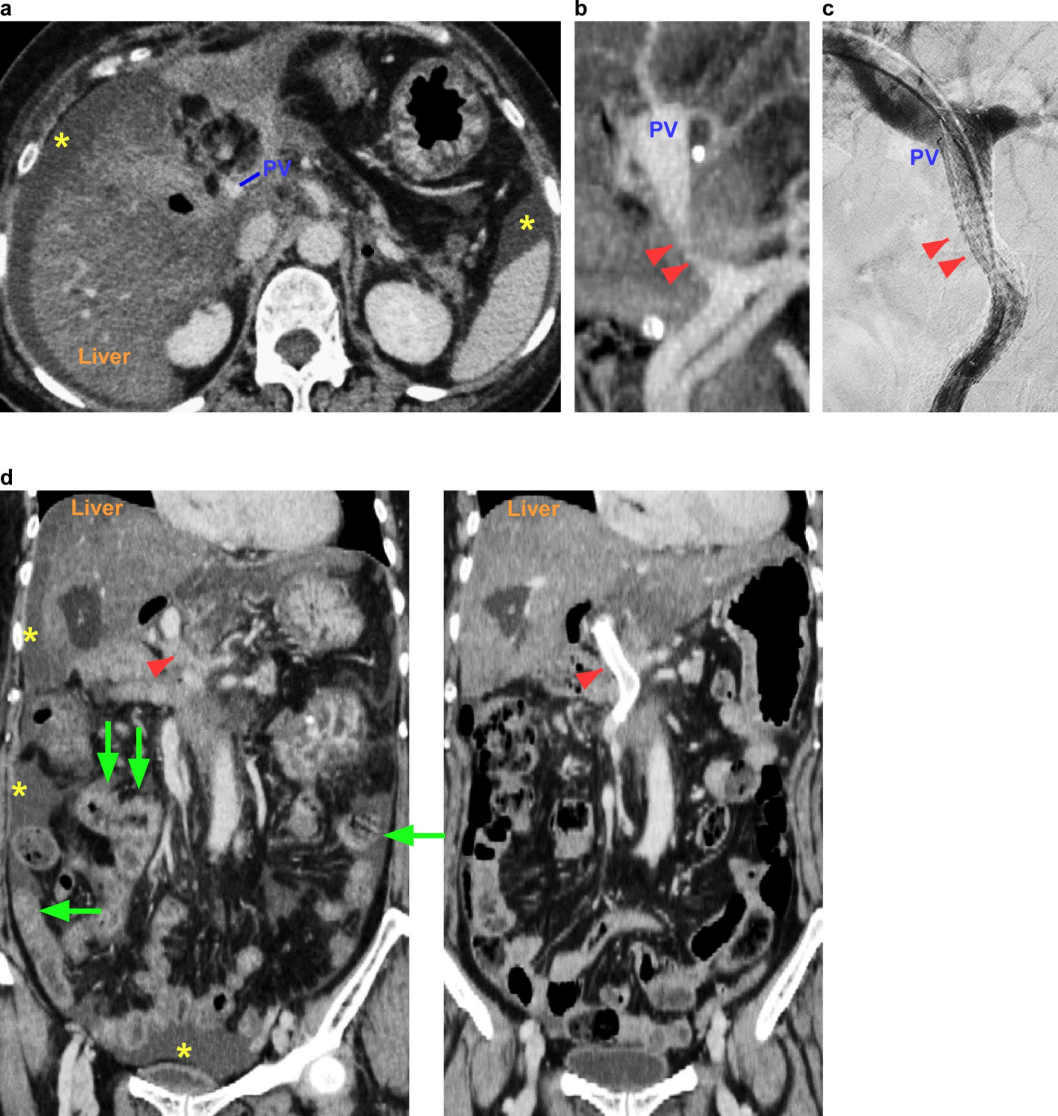
CT images on readmission and treatment for PV stenosis. **a** Liver CT values are decreased overall and there is ascites around the liver and spleen (_*_). **b** Maximum intensity projection image shows the PV stenosis (arrowhead). **c** PV stent is inserted via percutaneous transhepatic puncture. **d** Left: coronal CT image taken at the time of readmission showing ascites throughout and intestinal edema. Right: the same coronal CT view obtained 7 days after intervention shows that the ascites has disappeared and the intestinal edema has improved

A 70-year-old woman was referred to our hospital for investigation of epigastric discomfort and liver dysfunction. The patient had a history of hypertension and an overactive bladder. Her body mass index (BMI) was 29.3 kg/m^2^, and she had no metabolic diseases, such as diabetes or dyslipidemia. Laboratory data at the time of presentation are shown in Table 1. CT examination revealed a tumor in a distal bile duct (Fig. 1a); however, a tissue specimen could not be collected for diagnosis because of difficulty in approaching the common bile duct. Although the tumor was diagnosed as a cholangiocarcinoma using the rendezvous technique, the patient later developed pancreatitis (Fig. 1b). After receiving conservative treatment for pancreatitis and regaining her physical strength through rehabilitation, the patient underwent subtotal stomach-preserving pancreaticoduodenectomy. The inflammation around the PV was severe but could be resected without combined resection of the PV. The pathological specimen was a flat infiltrating mass that covered two-thirds of the bile duct lumen and was revealed to be a moderately to poorly differentiated adenocarcinoma, with negative resection margins. Based on the Union for International Cancer Control TNM staging, the pathological diagnosis was T1N1M0, stage IIA. Oral intake was started on the 6th postoperative day. The patient developed a fever on the 12th postoperative day and was found to have an infection from the drain, so antibiotics were administered. In addition, pancrelipase was started on the 19th postoperative day to treat diarrhea symptoms. After that, the postoperative course was stable and the patient was discharged on the 33rd postoperative day. However, the patient presented to the emergency department of our hospital with diarrhea and breathing difficulty on the 59th postoperative day. CT images showed marked fatty liver, ascites, intestinal edema, and PV stenosis (Fig. 2a, b). The next day, a PV stent was inserted by the percutaneous transhepatic approach and the PV flow improved (Fig. 2c). Although arterial bleeding from the liver was observed at the time of puncture, the bleeding spontaneously subsided after compression fixation. The intestinal edema and diarrhea symptoms also improved (Fig. 2d) and the patient was discharged on the 75th postoperative day with oral anticoagulants. At final follow-up 48 months after surgery, there were no signs of MASLD and no problems with the PV flow.

**Table 1:** Results of laboratory investigations

		First visit	Before surgery	POD 41	Readmission POD 59	POD 112	2 years after
Complete blood count							
WBC	× 10^2^/µl	45.2	62.6	58.3	60.7	38.9	45.6
RBC	× 10^4^/µl	464	414	386	405	431	431
Hb	g/dl	13.1	11.9	10.6	10.8	11.2	11.2
Plt	× 10^4^/µl	23.2	28.7	25.0	26.5	22.1	20.4
Neutr	/µl	2600	3500	3790	4200	2570	6390
Lypho	/µl	1420	2020	1410	1210	990	2630
Biochemistry							
T-Bil	mg/dl	6.84	1.17	0.44	0.56	0.44	0.36
AST	U/L	423	49	43	46	47	30
ALT	U/L	924	65	27	26	30	28
LDH	U/L	277	196	192	226	153	179
ChE	IU/L	-	-	184	156	237	329
TP	g/dl	6.7	6.8	6.4	6.4	6.9	7.2
Alb	g/dl	3.7	3.8	3.1	3.0	4.0	4.0
AMY	U/L	60	28	–	27	31	67
Lipase	U/L	102	63	–	31	21	48
T-CHO	mg/dl	182	208	136	134	165	136
TG	mg/dl	132	101	72	60	89	48
HDL-C	mg/dl	66.0	81.8	54.3	62.8	69.8	52.1
LDL-C	mg/dl	90	106.0	60.2	60.4	77.8	71.0
A1c	%	5.5	5.5	–	5.4	5.5	6.1
Zn	μg/dl	–	–	–	40	–	68
CRP	mg/dl	0.91	0.14	1.16	1.17	0.03	0.04
Coagulation							
PT	%	110.5	116.4	–	89.1	95	114.5

*WBC* white blood cells, *RBC* red blood cells, *Hb* hemoglobin, *Plt* platelets, *Neutr* neutrophil, *Lympho* lymphocytes, *T-Bil* total bilirubin, *AST* aspartate aminotransferase (GOT), *ALT* alanine aminotransferase (GPT), *LDH* lactase dehydrogenase, *ChE* cholinesterase, *TP* total protein, *Alb* albumin, *T-CHO* total cholesterol, *TG* triglyceride, *HDL-C* high-density lipoprotein cholesterol, *LDL-C* low-density lipoprotein cholesterol, *HbA1c* hemoglobin A1c, *Zn* zinc, *CRP* C-reactive protein, *PT* prothrombin time

## Discussion

The mechanism of the development of MASLD after PD is different from that of conventional MASLD. MASLD usually develops 4–12 months after surgery and may be due to three reasons [4]. First, the impaired pancreatic exocrine function is thought to be the main cause of MASLD after PD [5]. It has been reported that 56.3% of patients who undergo pancreatic surgery develop postoperative pancreatic exocrine insufficiency [8]. Obstructive pancreatitis and pancreatic atrophy occur due to pancreatic duct obstruction by tumors, resulting in decreased exocrine pancreatic function. As a result, impaired fat absorption leads to enhanced conversion of carbohydrates to fat in the liver [5]. Second, the endotoxins induce hepatic dysfunction due to diarrhea and gut barrier dysfunction with zinc deficiency [4, 5]. Intestinal mucosal atrophy is caused by diarrhea due to superior mesenteric artery plexus dissection and impaired pancreatic exocrine function, and bacterial translocation is induced [9]. Furthermore, zinc deficiency also induces diarrhea. Zinc binds to proteins contained in pancreatic enzymes and is absorbed in the duodenum and proximal jejunum, so zinc deficiency is common after PD [10]. As a result, endotoxins inflow into the liver via the PV, activating Kupffer cells and causing fatty deposition in the liver [11]. Third, postoperative malnutrition is suggested to be involved in the development of MASLD after PD [12]. PD is recognized as one of the most challenging operations because of the magnitude of the dissection and resection, and the resultant global stress. Malnourished patients or those who experience major complications after surgery may exhaust their nutritional reserves rapidly and thereby compromise their functional recovery and healing [13]. The decrease in the serum concentration of apolipoprotein B (a major component of very-low-density lipoprotein) caused by a diet deficient in choline induces the impaired hepatic export of triglycerides in the form of very-low-density lipoprotein [14]. As a result, there is a decrease in the export of fat to the outside of the liver, resulting in fatty deposition in the liver.

**Fig. 3:**
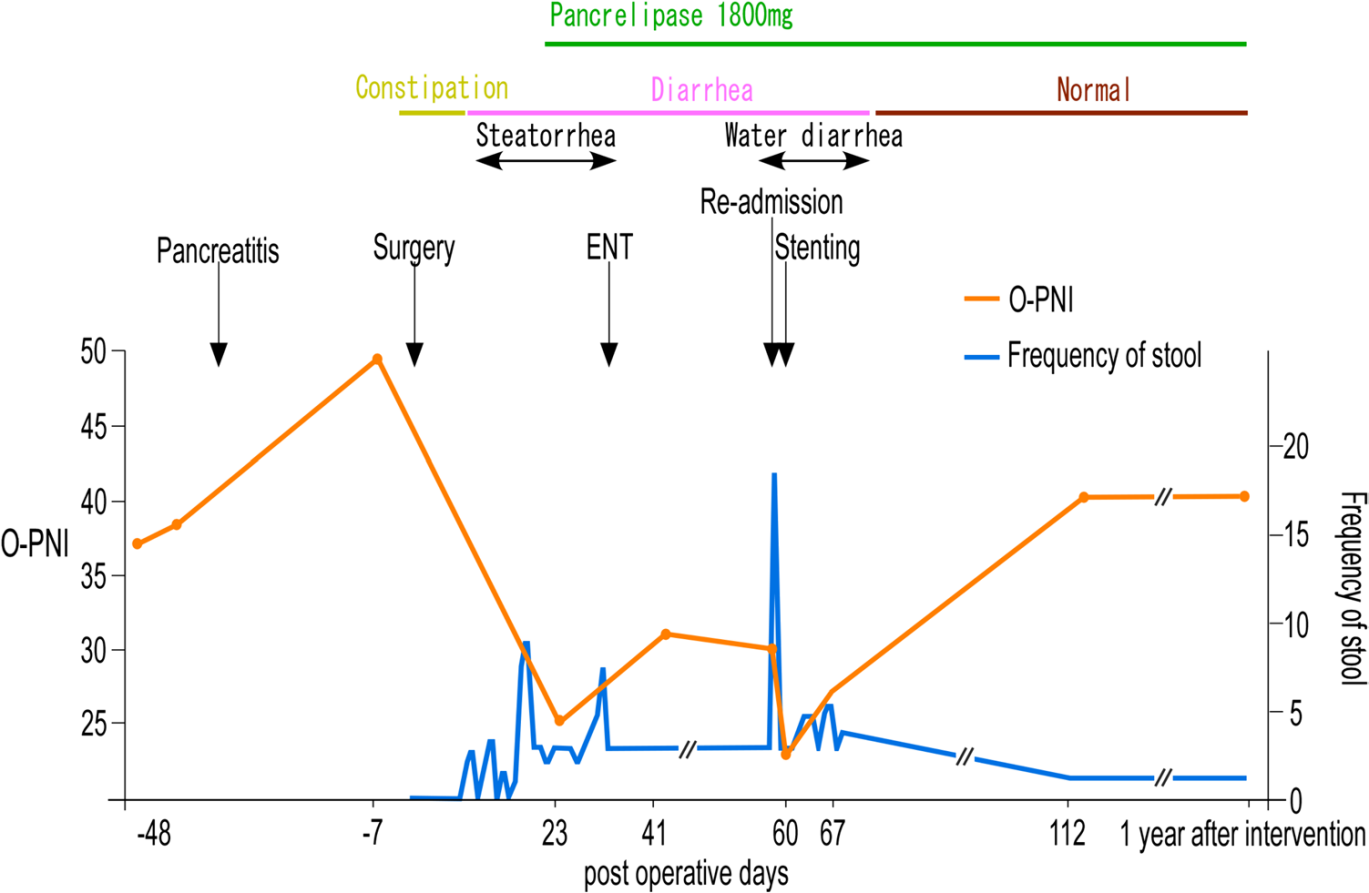
Time course of changes in diarrhea symptoms and nutritional markers. The frequency and nature of the stools and the changes in the onodera–prognostic nutritional index (O-PNI) are shown. The O-PNI temporarily worsened after PV intervention, but gradually recovered. The symptoms of diarrhea also gradually improved after intervention

**Fig. 4:**
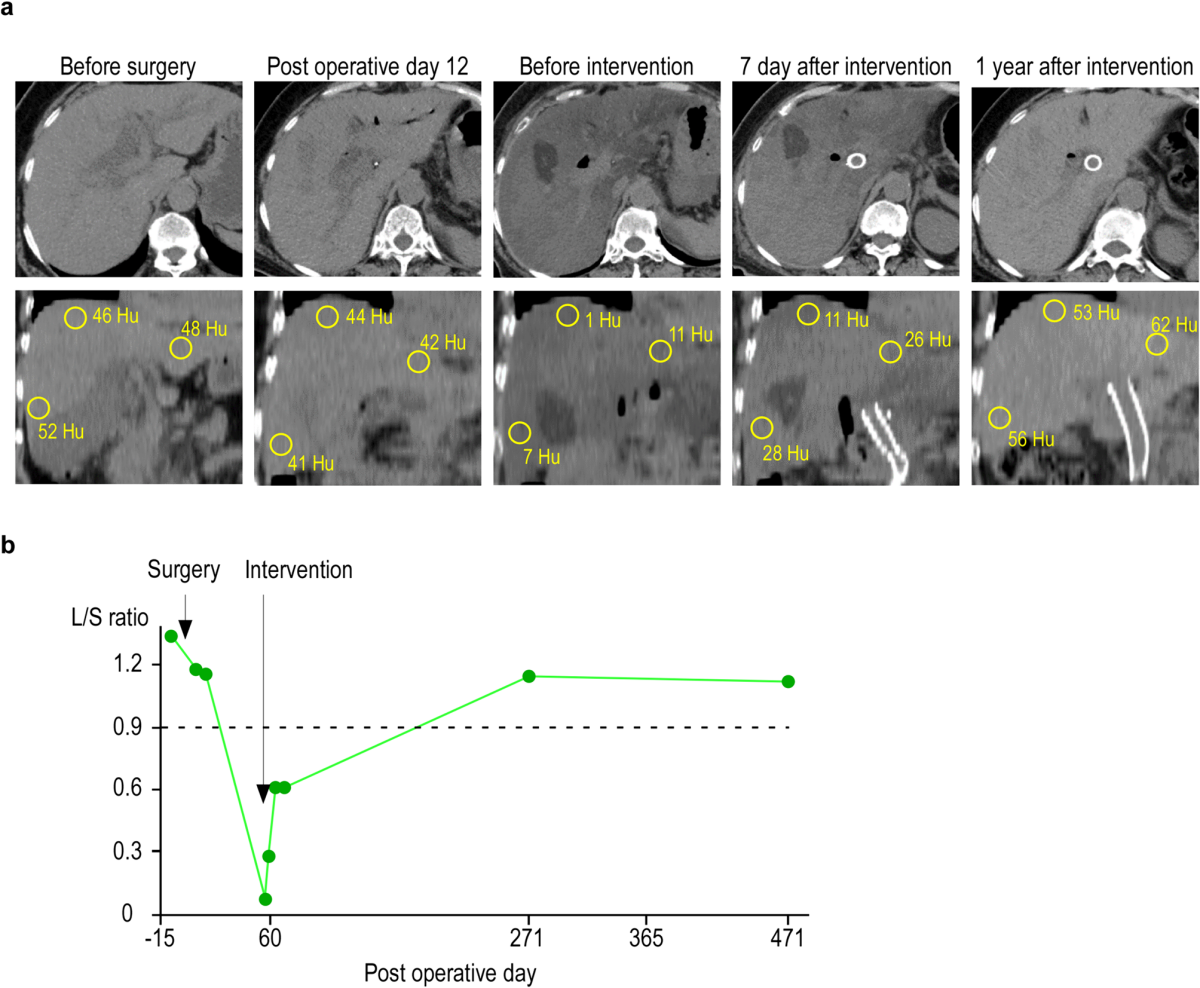
Changes in the CT images of the steatotic liver over time. **a** Axial and coronal views of the pre-contrast CT images over time show that the average CT values of the areas at S3, 5, and 8 are decreased due to the PV stenosis. **b** Average Hounsfield unit values of ten randomly selected parts of the liver and spleen show the gradual improvement in the liver-to-spleen (L/S) ratio after the intervention

In our case, SLD was rapidly progressed 2 months after surgery. There were no findings suggesting SLD on CT images obtained on postoperative day 12 or based on liver function tests performed after surgery (Table 1). Although a liver biopsy was not performed because of the risk of bleeding, CT imaging indicated the rapid deterioration of SLD. It has been suggested that an attenuation of 40 Hounsfield units on pre-contrast CT scans might be a practical cutoff value for predicting a liver fat content of 30% [15, 16]. Moreover, a liver-to-spleen ratio of less than 0.9 is considered an indicator of SLD [17, 18]. The SLD in the present case could have been caused by any of the reasons mentioned above. First, regarding nutritional disorders after surgery, the patient’s BMI was 27.3 kg/m^2^, which is considered obese in Japan and did not decrease after discharge, although she lost approximately 6% of her preoperative weight. Nutritional assessment using the Onodera–Prognostic Nutritional Index [19] showed a tendency toward recovery (Fig. 3). While her lymphocyte count was decreased and she had low levels of albumin and cholinesterase activity, these data appeared to have been within the scope of surgical invasion of PD because the patient was able to maintain her daily life activities. Considering past reports, it is unlikely that the SLD would rapidly worsen while the patient was in this nutritional state [12]. Second, the patient had pancreatitis before surgery and may have developed exocrine pancreatic insufficiency after surgery. The patient was taking pancrelipase for postoperative steatorrhea and this had improved by the time of discharge. In the days before her readmission, the patient had more than 10 bouts of diarrhea. At the time of readmission, the serum zinc concentration had decreased to 40 µg/dl. Considering the patient had pancreatic insufficiency, we initially considered increasing the doses of pancreatic enzymes and zinc supplements; however, we did not do so because if the coagulation function deteriorated further, transhepatic intervention would no longer be possible. Therefore, it was decided to prioritize treatment of the PV. As a result, the portal blood flow improved and the diarrhea symptoms were alleviated. After intervention, the pre-contrast CT values in the liver and the liver-to-spleen ratio returned to preoperative levels (Fig. 4). Considering that the stool quality had been stable and diabetes had not been an issue after intervention, it seems likely that exocrine pancreatic insufficiency was not the direct cause of the diarrhea. The profuse diarrhea might have been caused by small intestinal congestion due to PV stenosis, and it is thought that the SLD rapidly progressed due to impaired intestinal absorption and gut barrier dysfunction associated with zinc deficiency. After the PV intervention, the intestinal edema and L/S ratio on CT were soon improved (Figs. 2d and 4), and there has been no recurrence. The diarrhea symptoms and nutritional markers also gradually improved (Fig. 3). Although zinc levels did not be measured, there were no symptoms of hypozincemia, such as taste disorders. Then, it is considered that the SLD was alleviated by improvement of portal vein flow and absorption in the small intestine.

PV stenosis has a reported incidence of less than 20% after hepatobiliary surgery that involves resection of the PV and often occurs at 6 months after surgery [20]. The most common cause of PV stenosis is local tumor recurrence, especially pancreatic cancer, while benign stenosis is caused by inflammation of a pancreatic fistula [21, 22]. The early symptoms of PV stenosis are nausea, abnormal liver function test results, intestinal angina-like pain, and ascites, which are signs of portal hypertension [23]. As the pancreatic head, bile duct, and hepatoduodenal ligament are resected and it is difficult to form collateral veins to the liver, gastrointestinal bleeding is likely to occur [20]. In our case, the PV stenosis was thought to be caused by inflammation, as the preoperative pancreatitis was severe and a drain infection occurred after surgery. Ultrasonography confirmed that the flow of the intrahepatic PV was maintained; however, early treatment was deemed necessary because the disease was progressive. PV stenting is a safe and effective treatment for postoperative PV stenosis [24, 25].

In conclusion, we experienced a case of MASLD after PD in which the patient’s condition rapidly deteriorated with diarrhea and impaired intestinal absorption due to PV stenosis. MASLD after PD might develop for various reasons, so it is necessary to carefully monitor the postoperative recovery.
